# Influence of virtual reality and task complexity on digital health metrics assessing upper limb function

**DOI:** 10.1186/s12984-024-01413-x

**Published:** 2024-07-27

**Authors:** Christoph M. Kanzler, Tom Armand, Leonardo Simovic, Ramona Sylvester, Nadine Domnik, Antonia M. Eilfort, Carola Rohner, Roger Gassert, Roman Gonzenbach, Olivier Lambercy

**Affiliations:** 1 Rehabilitation Engineering Laboratory, Institute of Robotics and Intelligent Systems, Department of Health Sciences and Technology, ETH Zurich, Zurich, Switzerland; 2 Campus for Research Excellence And Technological Enterprise (CREATE), Future Health Technologies, Singapore-ETH Centre, Singapore, Singapore; 3 Rehabilitation Center Valens, Valens, Switzerland

## Abstract

**Background:**

Technology-based assessments using 2D virtual reality (VR) environments and goal-directed instrumented tasks can deliver digital health metrics describing upper limb sensorimotor function that are expected to provide sensitive endpoints for clinical studies. Open questions remain about the influence of the VR environment and task complexity on such metrics and their clinimetric properties.

**Methods:**

We aim to investigate the influence of VR and task complexity on the clinimetric properties of digital health metrics describing upper limb function. We relied on the Virtual Peg Insertion Test (VPIT), a haptic VR-based assessment with a virtual manipulation task. To evaluate the influence of VR and task complexity, we designed two novel tasks derived from the VPIT, the VPIT-2H (VR environment with reduced task complexity) and the PPIT (physical task with reduced task complexity). These were administered in an observational longitudinal study with 27 able-bodied participants and 31 participants with multiple sclerosis (pwMS, VPIT and PPIT only) and the value of kinematic and kinetic metrics, their clinimetric properties, and the usability of the assessment tasks were compared.

**Results:**

Intra-participant variability strongly increased with increasing task complexity (coefficient of variation + 56%) and was higher in the VR compared to the physical environment (+ 27%). Surprisingly, this did not translate into significant differences in the metrics’ measurement error and test–retest reliability across task conditions (p > 0.05). Responsiveness to longitudinal changes in pwMS was even significantly higher (effect size + 0.35, p < 0.05) for the VR task with high task complexity compared to the physical instrumented task with low task complexity. Increased inter-participant variability might have compensated for the increased intra-participant variability to maintain good clinimetric properties. No significant influence of task condition on concurrent validity was present in pwMS. Lastly, pwMS rated the PPIT with higher usability than the VPIT (System Usability Scale + 7.5, p < 0.05).

**Conclusion:**

The metrics of both the VR haptic- and physical task-based instrumented assessments showed adequate clinimetric properties. The VR haptic-based assessment may be superior when longitudinally assessing pwMS due to its increased responsiveness. The physical instrumented task may be advantageous for regular clinical use due to its higher usability. These findings highlight that both assessments should be further validated for their ideal use-cases.

**Supplementary Information:**

The online version contains supplementary material available at 10.1186/s12984-024-01413-x.

## Introduction

Upper limb disability is common in neurological disorders, such as persons with multiple sclerosis (pwMS), which strongly contributes to an inability to perform daily life activities and increases dependency on caregivers [1]. In clinical studies, *assessments* are of fundamental importance to advance our understanding of the types of upper limb impairments and their underlying mechanisms [2]. In addition, assessments are essential to provide sensitive and reliable endpoints that can be used to evaluate the effectiveness of pharmacological or rehabilitation interventions.

The most commonly applied assessments in clinical studies subjectively describe movement quality on ordinal scales or capture the time to complete functional tasks [2]. While these assessments have high usability, provide a good overview of the disability level of a patient, and are well-accepted by the clinical community, they have a limited ability to serve as detailed, insightful endpoints for clinical studies [2, 3]. This is because ordinal scales typically have ceiling effects and low sensitivity, while subjective assessments are prone to rater-induced bias [4]. In addition, time-based assessments are not able to provide information on the mechanism underlying suboptimal task performance; for example, they cannot distinguish whether grip force control or gross movement control is impaired. Because of these limitations, there is a consensus in the research community that novel, complementary and more sensitive endpoints are urgently required to provide more detailed insights into the mechanisms of upper limb impairments and the effect of therapeutic interventions [3, 5, 6].

Technology-based assessments can record objective sensor-based data on upper limb movement patterns and hand grip forces during functional manipulation tasks [7, 8]. These can be transformed into digital health metrics (discrete one-dimensional metrics extracted from health-related sensor data such as movement kinematics and kinetics) with ratio scales, thereby promising novel, sensitive, and insightful endpoints [9, 10]. Technology-based assessments often consist of a robotic interface (e.g., haptic devices) that serves as a control input (i.e., joystick) and a virtual reality (VR) environment with a goal-directed manipulation task rendered, for example, on a 2D computer screen [11], [12–15].

VR environments are a unique element of technology-based assessments, as they provide flexibility in the implementation of assessment tasks with different levels of complexity to target specific sensorimotor and cognitive impairments. Also, VR environments promise to increase engagement and motivation of participants, and VR-based depth cues can support a realistic representation of 3D movements on a 2D screen [16, 17]. However, when compared to physical environments, VR environments and the different levels of task complexity they may generate are also known to influence the kinematics of goal-directed movements. This can be, for example, in terms of reduced smoothness and speed, or increased movement variability [15, 18–22]. Crucially, it remains an open question whether this change in kinematics and variability also influences the extracted digital health metrics and in particular their *clinimetric properties*. These properties include test–retest reliability, measurement error, responsiveness, and concurrent validity and ultimately determine whether digital health metrics can be used as insightful and robust endpoints in clinical studies [9, 10, 23].

The aim of this work is to describe the influence of VR and task complexity on the clinimetric properties of digital health metrics extracted from a goal-directed, technology-based upper limb assessment. The secondary aim is to describe the influence of these two factors on the magnitude of the metrics, the observed intra-participant variability, and the usability of the technology-based assessment.

**Fig. 1:**
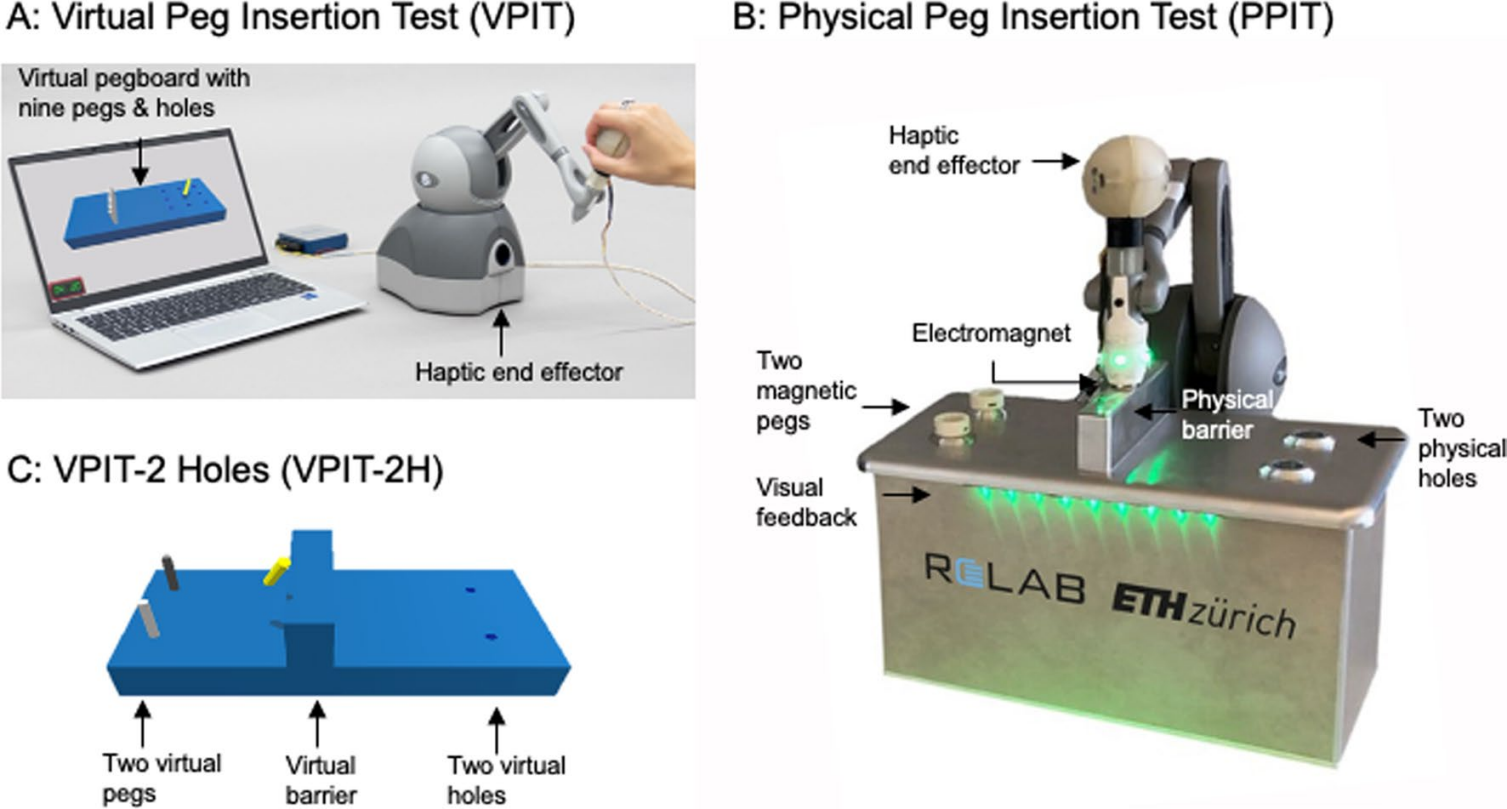
Assessment platforms VPIT (**A**), PPIT (**B**), and virtual display of the VPIT-2H (**C**). These are used to study the influence of task complexity and virtual reality on the clinimetric properties of digital health metrics

**Fig. 2:**
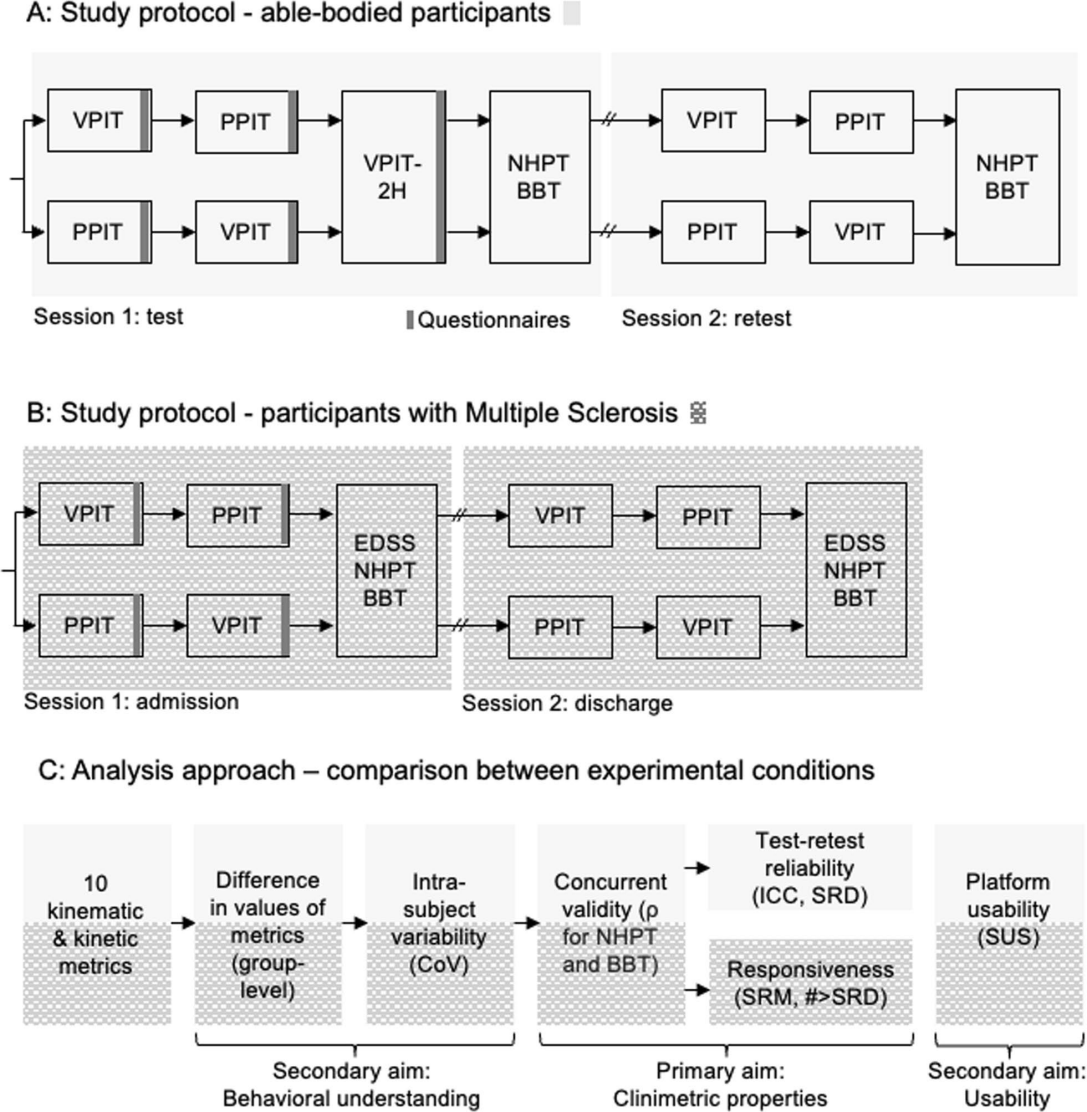
Overview of study protocol (**A**, **B**) and analysis approach (**C**). Able-bodied participants were tested on the VPIT, PPIT, and VPIT-2H in a test–retest protocol. Participants with Multiple Sclerosis were tested with the VPIT and PPIT at admission and discharge within a 3-week neurorehabilitation program. The analysis focused on a comparison between the three experimental conditions (VPIT, PPIT, VPIT-2H) for the clinimetric properties (primary aim) and to gain a behavioral understanding of the effect of experimental conditions and describe the usability of the assessments (secondary aim). EDSS: Expanded Disability Status Scale. NHPT: Nine Hole Peg Test. BBT: Box and Block Test. CoV: Coefficient of Variation. ICC: Intra-class correlation coefficient. SRD: Smallest Real Difference. SRM: Standardized response mean. # > SRD: Number of individuals with changes larger than the SRD. SUS: System Usability Scale

For this purpose, we relied on the Virtual Peg Insertion Test (VPIT, Fig. 1), a previously established haptic end effector- and VR-based assessment describing upper limb movement patterns and hand grip force control during the insertion of nine virtual pegs into nine holes. To assess the impact of both task complexity and VR, we developed two distinct assessment tasks: the VPIT-2 Hole (VPIT-2H, Fig. 1) requires inserting only two virtual pegs into two virtual holes, thereby simplifying the original VPIT. To examine the influence of VR, we introduced the Physical Peg Insertion Test (PPIT, Fig. 1). The PPIT uses the same end effector device as the VPIT, but instead of a virtual task, it uses a physical pegboard with two magnetic pegs and physical holes, and an electromagnet to transport the magnetic pegs. These assessments were administered in an observational longitudinal study with 27 able-bodied participants (VPIT, VPIT-2H, and PPIT; test and retest) and 31 pwMS (VPIT and PPIT; admission and discharge to a rehabilitation program; Fig. 2).

We hypothesized that a physical technology-based assessment task reduces intra-participant variability when compared to a similar VR-based task, where a complex visuomotor mapping is required to match position of the end-effector and the non-collocated VR environment. Similarly, we expected that decreasing task complexity reduces the observed intra-participant variability. Additionally, we expected that this reduced intra-participant variability leads to increased test–retest reliability and responsiveness as well as reduced measurement errors in the extracted digital health metrics. Lastly, we hypothesized that a physical environment has higher concurrent validity and usability, as it more closely resembles the tasks of conventional clinical scales.

Addressing these research questions would provide evidence that can inform the future design and choice of technology-based assessments for sensitively and robustly monitoring upper limb impairments in clinical studies.

## Methods

### Participants and procedures

This observational, longitudinal study was performed at two sites, namely ETH Zurich (Zurich, Switzerland) where able-bodied participants were recruited and the Rehabilitation Centre Valens (Valens, Switzerland) where pwMS were recruited. For able-bodied participants, the inclusion criteria were age of at least 18 years and the ability to follow procedures and to give informed consent. All pwMS being admitted to the Rehabilitation Center Valens for a 3-week inpatient rehabilitation program focused on achieving individualized patient goals were screened for eligibility based on the standard physical examination protocol of the clinic. Patient goals did not necessarily need to include upper limb function and were defined in agreement with clinical personnel and patient. Inclusion criteria were a confirmed diagnosis of MS, age of at least 18 years, and the ability to follow procedures and to give informed consent. Additionally, pwMS were required to have mild to moderate upper limb disability and an absence of strong cognitive deficits. This was evaluated based on the standard medical report generated at clinic admission through the absence of, for example, strong paresis, spasticity, or neglect. If no relevant information on cognitive function or upper limb disability was available in the report, an experienced clinical researcher of the study team would subjectively evaluate the participant. For both populations, exclusion criteria were concomitant diseases that affect upper limb function.

Able-bodied participants performed an initial assessment session followed by a break of three weeks and another retest session. Each session consisted of the VPIT, PPIT, and VPIT-2H protocol performed with the dominant hand. PwMS participated in one assessment session upon admission to the rehabilitation centre and one session before discharge (Fig. 2). Each session consisted of the VPIT and PPIT protocol, questionnaires, and conventional clinical assessments performed with one body side. The most suitable body side for the assessment was chosen based on the disability level of the patient. To avoid fatigue, the VPIT-2H was not performed in pwMS. No additional test–retest session was scheduled for pwMS to avoid extra burden on participants. For both populations, the order of performing the VPIT or PPIT was pseudo-randomized.

All study procedures were approved by the respective ethics commissions (EKOS 21/045).

### Technology-based assessments: VPIT, VPIT-2H, and PPIT

The VPIT is a well-established technology-based assessment of upper limb movement patterns and hand grip forces, which has been extensively applied and validated in able-bodied participants and persons with neurological disorders [9, 10, 24, 25]. In brief, the approach relies on a haptic end effector (Phantom Omni or Geomagic Touch, 3D Systems, US), a custom-made handle with integrated force sensors, and a computer displaying a 2D projection of a VR environment. The VPIT task consists of a virtual pegboard and nine virtual pegs that need to be inserted as fast and accurately as possible into nine corresponding holes. Pegs can be picked up in arbitrary order and transported into any of the holes. Initially, the virtual cursor needs to be lifted to the base height of the virtual pegboard. To pick up and transport a peg, the cursor needs to be spatially aligned with the peg and a grip force of at least 2N must be applied until insertion into a hole. No requirements or instructions are given to increase vertical displacement during peg transport. If a grip force of at least 2N is applied before cursor and peg are spatially aligned, the color of the cursor changes to red. If a peg is successfully lifted, the color of the cursor becomes green. While the VPIT may visually resemble the conventional Nine Hole Peg Test (NHPT), the VPIT requires different movements (i.e., arm lifting and gross upper limb movement) as well as a different grip (i.e., power grasp) on the handle. Thus, it should be interpreted as a hybrid assessment between the NHPT and the Box and Block Test (BBT), testing both fine and gross manual dexterity and fine power grasp control. The typical VPIT protocol consists of a standardized seating position and standardized instructions, an initial familiarization period where the participant can explore the VR environment, followed by five repetitions of the task. Herein, we applied a shortened version of the protocol with only three task repetitions (i.e., inserting nine pegs three times) that has shown a good trade-off between applicability and robustness [25].

To evaluate the influence of task complexity, an additional VR task based on the VPIT concept but with reduced complexity was designed (VPIT-2H, Fig. 1). In the VPIT-2H, only two instead of nine pegs are displayed and need to be transported into corresponding holes. In addition, each peg needs to be inserted into the hole that is aligned with the initial position of the peg. The task needs to be started with picking up the peg that is closer to the participant, of the two available pegs. This aims to increase standardization to reduce intra-participant variability and ultimately improve clinimetric properties of kinematic and kinetic metrics. Further, a haptically rendered virtual wall was placed on the pegboard to force the vertical lifting of the cursor and arm during the task and facilitate more natural movements. Equivalent to the VPIT, the VPIT-2H protocol starts with standardized instructions and an initial familiarization period. Subsequently, 14 task repetitions are performed (i.e., inserting two pegs 14 times; 28 transport movements) to match the number of movements performed in the regular VPIT (27 transport movements over three repetitions).

To further evaluate the influence of VR, a physical task similar to the VPIT-2H was designed, the PPIT (Fig. 1). The PPIT relies on the same haptic end-effector and grip force sensing handle as the VPIT and the same task as the VPIT-2H but has no VR component. Importantly, an electromagnet that is controlled by the applied grip forces was attached at the bottom of the end-effector, allowing to “grasp” the physical pegs through the handle of the haptic device. Additionally, a magnet was included in the physical pegs such that they can be picked up by the electromagnet. Infrared through-beam sensors were placed in all holes to capture whether a physical peg has been lifted and inserted into a hole. Additionally, LED stripes providing feedback on the status of the electromagnet were added to the sides of the pegboard. The LED lighted up in green if at least 2N of grip force were applied to match the requirements of the VPIT. In addition, the LEDs lighted up in red when 5N grip force were exerted to avoid handle damage. Equivalent to the VPIT-2H, the PPIT protocol consists of standardized instructions, an initial familiarization period, and 14 task repetitions (i.e., inserting two pegs 14 times). The dimensions of the pegboard and pegs of the VPIT-2H and PPIT were designed to approximate the ones of the VPIT to ensure comparability between the movements in different conditions.

While there is no alignment in the research community about how to best describe task complexity [26], we relied for our definition on the concept of component complexity [27], which focuses on the number of distinct actions and information cues required for task performance. In this study, component complexity is reflected by the increased number of actions that need to be executed to perform one repetition of the task in the VPIT as compared to the VPIT-2H and PPIT. Additionally, the number of information cues that need to be processed is considerably higher in the VPIT compared to the VPIT-2H and PPIT given that the former features nine instead of two pegs and does allow flexibility in the order in which the pegs and holes are chosen. Moreover, the VPIT and VPIT-2H require learning a complex spatial transformation from the end-effector to the VR coordinate system, as opposed to the PPIT that has end-effector and task coordinate system physically aligned, thus further reducing the complexity of the PPIT. Another design feature potentially influencing task complexity is the presence of a wall requiring cursor lifting during peg transport in the VPIT-2H and PPIT but not the VPIT condition. While the same type of grip is used for VPIT, VPIT-2H, and PPIT, different visual feedback of the applied grip force is provided in the VR (feedback via screen) and physical condition (feedback via LEDs). This is however not expected to systematically influence task complexity.

### Extraction of digital health metrics

Based on previous work with the VPIT in able-bodied and neurological participants, a core set of 10 validated digital health metrics describing the most important aspects of movement patterns and hand grip forces has been defined [9, 10, 25, 28]. While these metrics have been refined through additional studies [25], we herein keep the initial 10 core metrics to best cover different behavioral aspects of the test in view of the comparison of different experimental conditions. Given that the VPIT, VPIT-2H, and PPIT collect the same type of movement and grip force data and task dimensions are matched across conditions, the signal processing framework that was initially defined for the VPIT could be seamlessly applied to the VPIT-2H and PPIT. In the following, we will briefly restate the signal processing steps and the definition of the metrics, whereas details are provided in previous work [9, 10].

First, the position and grip force time-series recorded by the haptic device at a sampling rate of 1 kHz were pre-processed using standard low-pass filtering and interpolation operations. Second, the time-series were temporally segmented into multiple phases that engage different aspects of motor control. This included the *transport* phase (i.e., ballistic movement between lifting a peg and inserting it into a hole), the *return* phase (i.e., ballistic movement between inserting a peg into a hole and lifting the next peg), the *peg approach* phase (i.e., fine movement before lifting a peg), and the *hole approach* phase (i.e., fine movement before inserting a peg). Third, digital health metrics were extracted to describe different aspects of movement patterns and grip forces. More specifically, the logarithmic normalized jerk (*log jerk transport/return*) as well as the spectral arc length (*SPARC return*) metrics were calculated to describe movement smoothness [29]. The SPARC metric captures the normalized arc length of the velocity spectrum. A short arc length reflects a spectrum with few dominant frequency components (i.e., submovements) and is indicative of smooth movements [29]. Further, the ratio between the shortest possible and the actual path in the horizontal plane (*path length ratio transport/return*) was used to capture movement efficiency [30]. In addition, the maximum velocity during the *return* phase (*max. velocity return*) was calculated to describe the speed of ballistic movements. To collect information on fine position adjustments when picking up pegs, the jerk metric was calculated during the *peg approach* phase (*jerk peg approach*). Lastly, hand grip force control was characterized based on the number of peaks in the grip force rate profile (*force rate num. peaks transport*) and the spectral arc length of the grip force rate (*force rate SPARC transport/hole approach*) [31].

For each assessment task, the metrics were calculated on a peg-by-peg level and then aggregated via the grand median to obtain one value per session and body side. Compared to the regular VPIT processing pipeline, we did not normalize the metrics with respect to an able-bodied population given that such data is not available for VPIT-2H and PPIT [9, 10].

### Conventional clinical assessments and questionnaires

Two conventional clinical assessments were performed with all participants to allow comparing the concurrent validity of the digital health metrics between the different technology-based assessment tasks. Gross manual dexterity was assessed using the BBT, which describes the number of wooden blocks that can be transported over a physical barrier within one minute [32, 33]. Also, fine manual dexterity was captured with the NHPT which describes the time to transport nine physical pegs into nine physical holes and is a well-accepted outcome measure in pwMS [32–34]. Additionally, the overall disability level of pwMS was rated based on the Expanded Disability Status Scale (0: no disability; 10: death due to MS) [35].

Furthermore, all participants were asked to perform the System Usability Scale (SUS) after the completion of each technology-based assessment task during the first measurement session. The SUS is a well-accepted 10-item usability questionnaire describing effectiveness, efficiency, and satisfaction of a system and ranges from 0 (worst usability) to 100 (best usability).

### Data analysis

The analysis steps described below were performed separately for each of the available technology-aided assessments. Afterwards, the outcomes were compared between assessments using statistical tests, namely a Wilcoxon signed rank test or Friedman omnibus test for non-parametric paired samples, followed by post-hoc tests (MATLAB version R2022b, functions *signrank, friedman* and *multcompare*).

#### Usability

Usability of the technology-based assessments was evaluated based on the SUS. Scores above 71.1 were interpreted as ‘acceptable’ usability [36].

#### Intra-subject variability

In order to have endpoints that are reliable, have low measurement error, and are sensitive, it is essential that participants have low variability when repeating the task within an assessment session (i.e., low intra-participant variability) [37, 38]. To evaluate intra-participant variability, we calculated the coefficient of variation, which is defined as the standard deviation of a metric divided by the absolute value of its mean, for each metric and participant. For this analysis, the cross-sectional data from the first measurement timepoint were used on a peg-by-peg basis (i.e., one value per peg) to account for the difference in number of movements per repetitions across task and take the intra-participant variability into account.

#### Test–retest reliability

Test–retest reliability takes the intra- and inter-participant variability into account and describes the ability of a metric to discriminate across participants and measurement sessions [9, 10, 37, 38]. In simplified terms, test–retest reliability can be expressed as $reliability = \frac{inter-participant\ variability}{inter-participant\ variability\ +\ error}$, where error includes any source of systematic or random error, including intra-participant variability [38]. Test–retest reliability is commonly described using the agreement intra-class correlation coefficient (ICC, 0: worst reliability, 1: best reliability), which was calculated based on a two-way analysis of variance (ICC A,1) [37]. For this analysis, the test–retest data from able-bodied participants were used and aggregated per session (i.e., one value per session).

#### Measurement error

The measurement error describes a range of values for which the assessment is not able to discriminate between measurement-related noise, for example due to high intra-participant variability, and an actual physiological change [9, 10, 37]. Mathematically, this is expressed as the *smallest real difference*, which is dependent on the intra-participant variability ($\Sigma )$ and the ICC: $SRD=1.96 \sqrt{2}\Sigma \sqrt{1-ICC}$ [37]. To allow a comparison between metrics, the SRD can further be normalized with respect to the range of observed values (SRD%) [9, 10]. For this analysis, the test–retest data from able-bodied participants were aggregated per session.

#### Concurrent validity

Even though digital health metrics are expected to provide information complementary to conventional clinical scales, one can still expect at least low to moderate correlations between digital health metrics and conventional clinical scales capturing similar physiological constructs [7]. To evaluate concurrent validity, we calculated Spearman correlation coefficients (ρ) between digital health metrics and selected clinical scales that assess similar constructs as the VPIT, namely the BBT and NHPT. We used the absolute value of the correlation coefficient to allow an analysis across metrics where different signs of the correlation might indicate a positive effect. For this analysis, cross-sectional data from the first assessment session were aggregated.

#### Responsiveness

Responsiveness denotes the ability of a metric to capture intervention-induced changes [39]. To describe responsiveness, we counted the number of pwMS that exhibited a meaningful change according to the digital health metrics. This was defined as a change in a digital health metric between admission and discharge that exceeded the SRD, which is an accepted measure of responsiveness [37]. Additionally, we used the standardized response mean (SRM), which is the difference in means between admission and discharge divided by the standard deviation of changes between admission and discharge, to describe the population-level effect sizes [39]. The level of the effect can be broadly categorized into small (0.2 ≤ SRM < 0.5), moderate (0.5 ≤ SRM < 0.8), and high (SRM ≥ 0.8). Also, to compare the responsiveness of the digital health metrics to the clinical level of responsiveness, we calculated additionally the SRM for the NHPT and BBT. Lastly, we counted the number of metrics that indicated a statistically significant change across the rehabilitation program. For this analysis, only pwMS were included that had complete data at admission and discharge, and the data were aggregated per session.

## Results

Participant details are provided in Table 1. In brief, 27 able-bodied participants (age 30.5 ± 15.5 years, 15 male, reported as median ± interquartile range) were recruited and completed the assessment protocol at the first testing session. Fifteen of those further participated in a second retest session. Further, 34 pwMS were recruited. Of those, 31 (age 56 ± 19.5 years, 16 male, EDSS 4.5 ± 3.5) completed the assessment session at admission to the rehabilitation program, whereas 21 completed the assessment session at discharge. Reasons for participants not completing the assessment protocol were: too severe upper limb disability, unexpected discharge from the rehabilitation program, and technical difficulties with the assessment platforms.

**Table 1:** Participant details

Able-bodied participants	Test	Retest
n	27	15
Age	30.5 ± 15.5 (21–68)	34 ± 24.5 (23–68)
Sex	15 m, 12 f	9 m, 6 f
Box and Block Test (blocks per minute)	77.0 ± 8.5 (66.0–92.0)	–
Nine Hole Peg Test (s)	17.0 ± 3.9 (12.1–23.0)	–

Participants with multiple sclerosis	Admission	Discharge
n	31	21
Age	56 ± 19.5 (33–72)	57 ± 21 (34–69)
Sex	16 m, 15 f	13 m, 8 f
Multiple sclerosis type	6 primary progressive 12 secondary progressive 13 relapsing–remitting	4 primary progressive 7 secondary progressive 10 relapsing–remitting
Expanded Disability Status Scale (0–10)	4.5 ± 3.5 (2.5–6.5) 1 missing value	4.75 ± 3.25 (2.5–6.5) 1 missing value
Box and Block Test (blocks per minute)	54.0 ± 22.0 (18.0–70.0) 7 missing values	55.0 ± 24.0 (22.0–70.0) 8 missing values
Nine Hole Peg Test (s)	30.6 + −17.0 (16.2–160)	30.7 + −29.5 (16.3–94.3)

The content of each row is described in the first table column. Values are denoted as median ± interquartile range (minimum–maximum)

### Influence of task setup and complexity on kinematics and kinetics

While the main objective of this work is to evaluate the impact of task conditions on the clinimetric properties, we had to first confirm previous reports about the impact of VR and task complexity on movement kinematics and kinetics. Thus, the results for this are summarized here, whereas the details are provided in Figures SM1–4.

**Fig. 3:**
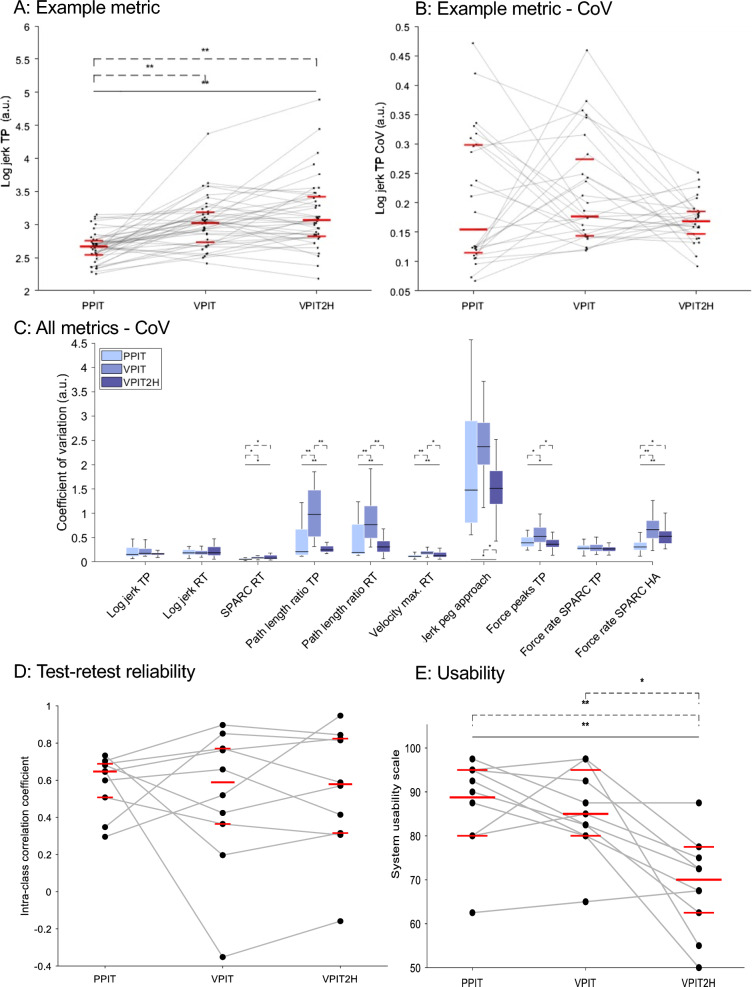
Able-bodied participants. Visualization of an example metric across all conditions (**A**, grey lines connect individual participants), its coefficient of variation (CoV, **B**, grey lines connect individual participants), and the CoV for all metrics and conditions (**C**). In addition, the test–retest reliability of metrics across all conditions (**D**, grey lines connect individual metrics) and the usability outcomes (E, grey lines connect individual participants) are depicted. The middle, long horizontal bar represents the median and the shorter horizontal bars or end of the filled box the 25th- and 75th-percentile. The whiskers in C represent the minimum and maximum value within 1.5 times the interquartile range. A.u. arbitrary units. *p < 0.05. **p < 0.01

In brief, for able-bodied participants, goal-directed movements were statistically smoother and faster in the PPIT than the VPIT and VPIT-2H according to the *log jerk transport* and *return, velocity max. return* metrics (Fig. 3A). Additionally, movements were more efficient in the VPIT than the VPIT-2H and PPIT according to the *path length ratio transport* and *return* metrics. Further, fine movements when approaching a peg were jerkier for the PPIT than the VR-based tasks according to the *jerk approach peg* metric*.* Moreover, grip force control was smoother in able-bodied participants for the PPIT and VPIT than the VPIT-2H according to *force rate number of peaks transport* and *force rate SPARC transport* metrics. Lastly, grip force control when approaching a hole was significantly smoother for the PPIT compared to the VPIT and VPIT-2H according to the *force rate SPARC hole approach* metric.

**Fig. 4:**
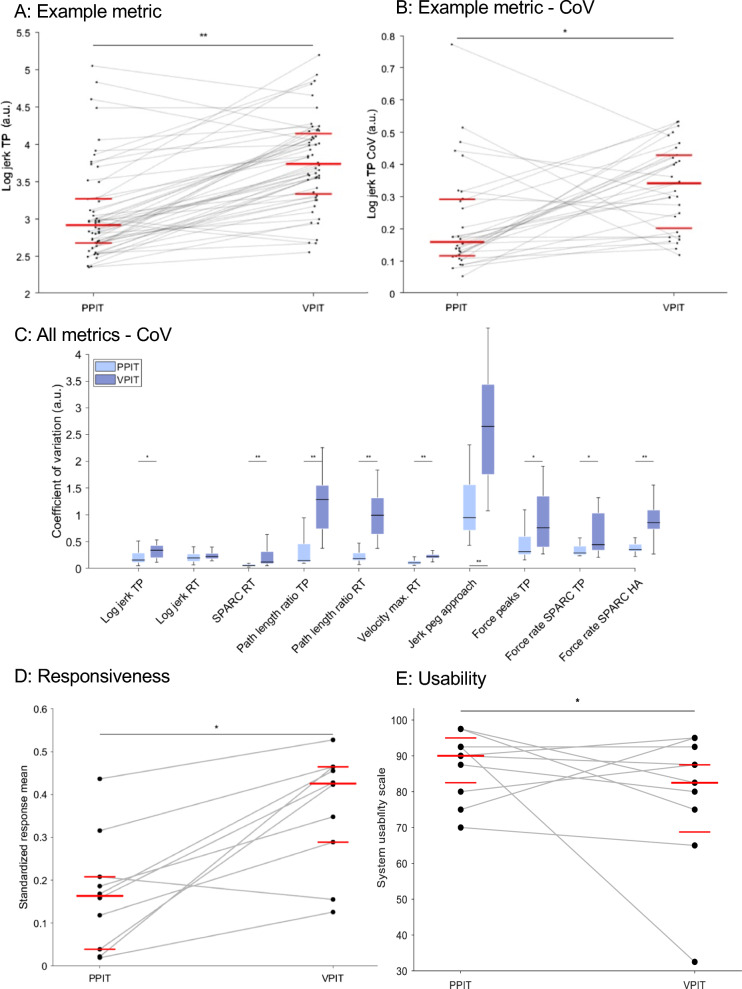
Participants with multiple sclerosis. Visualization of an example metric for all participants with multiple sclerosis across all conditions (**A**), its coefficient of variation (CoV, **B**), and the CoV for all metrics and conditions (**C**). In addition, the responsiveness of all metrics across all conditions (**D**) and the usability outcomes (**E**) are depicted. Detailed legend in Fig. 3

For pwMS (Fig. 4A), goal-directed movements were significantly smoother for the PPIT than the VPIT according to the *log jerk transport* and *return* metrics. These trends were confirmed, but not significant, for the *SPARC return metric.* Movements were significantly more efficient for the VPIT than the PPIT according to the *path length ratio transport* and *return* metrics. Movements were significantly faster for the PPIT than the VPIT according to the *velocity max. return* metric. No significant difference between VPIT and PPIT in the jerkiness of movements when approaching a peg were found. Force control during goal-directed movements did not differ significantly between conditions, whereas force control when approaching a hole was smoother for the VPIT than the PPIT.

### Intra-participant variability

For the intra-participant variability in able-bodied participants (Fig. 3B and C), the coefficient of variation was significantly smaller for the PPIT (0.20 ± 0.15 across all metrics) than the VPIT (0.40 ± 0.58 across all metrics) for all metrics except *log jerk transport and return, jerk peg approach* and *force rate SPARC transport*. In addition, the *path length ratio transport* and *return,* the *velocity max. return*, the *jerk peg approach*, and the *force rate number of peaks transport* metrics had significantly smaller coefficient of variations for the VPIT-2H (0.26 ± 0.20 across all metrics) than the VPIT. The *SPARC* return and the *velocity max. return* metrics indicated significantly smaller coefficients of variation for the PPIT than the VPIT-2H. For the intra-participant variability in pwMS (Fig. 4B and C), the coefficient of variation was significantly smaller for the PPIT (0.19 ± 0.17) than the VPIT (0.60 ± 0.77) for all digital health metrics except for *log jerk return*.

### Clinimetric properties

For the test–retest reliability in able-bodied participants, the ICC across metrics for the PPIT, VPIT, and VPIT-2H was 0.64 ± 0.18, 0.66 ± 0.35, and 0.58 ± 0.43, respectively (difference between groups p > 0.05, Fig. 3D). The ICC and confidence interval for individual metrics is shown in Figure SM5: confidence intervals of the ICC were large across most metrics and the most notable difference in ICC between conditions was for the *SPARC return*, where VPIT-2H and VPIT performed considerably better than the PPIT.

For the measurement error in able-bodied participants, the SRD% was 39.4 ± 12.3 for the PPIT, 36.9 ± 34.4 for the VPIT, and 38.8 ± 21.7 for the VPIT-2H (difference between groups not significant p = 0.74). The SRD and SRD% for all metrics is listed in Table SM1 in the Supplementary Materials (SM).

In terms of responsiveness (details in Table 2, Fig. 4D, and SM), the VPIT was superior to the PPIT according to the number of pwMS exhibiting changes above the SRD (increased by 2.5, p > 0.05), effect sizes (SRM increased by 0.35, p < 0.05), and number of metrics indicating significant changes over the rehabilitation program (4 for the VPIT indicating improvement in upper limb function, 1 for the PPIT). Responsiveness for the NHPT and BBT are described in the SM.

**Table 2:** Responsiveness (standardized response mean SRM and number of individuals with changes exceeding the measurement noise # > SRD) and concurrent validity (correlation ρ to Nine Hole Peg Test NHPT and Box and Block Test BBT) for persons with MS

Metric	PPIT				VPIT			
	SRM	# > SRD	ρ BBT	ρ NHPT	SRM	# > SRD	ρ BBT	ρ NHPT
Log Jerk TP	− 0.32	5	− 0.84	0.68	− 0.46	1	− 0.56	0.63
Log Jerk RT	0.19	4	− 0.62	0.75	− 0.35*	0	− 0.58	0.67
SPARC RT	− 0.12	7	− 0.50	0.44	− 0.29	10	− 0.67	0.79
Path length ratio TP	0.02	5	− 0.14	0.27	− 0.13	7	− 0.45	0.32
Path length ratio RT	− 0.17	3	− 0.49	0.45	− 0.46*	7	− 0.49	0.32
Velocity max. RT	0.21	0	0.48	-0.65	0.16	3	0.53	− 0.56
Jerk peg approach	0.16*	8	− 0.50	0.67	− 0.43	10	− 0.38	0.28
Force rate num. peaks TP	− 0.44	2	− 0.57	0.52	− 0.53	5	− 0.42	0.44
Force rate SPARC TP	0.02	1	− 0.53	0.41	− 0.47*	1	− 0.34	0.50
Force rate SPARC hole approach	0.04	3	− 0.48	0.39	− 0.42*	8	− 0.57	0.47
Aggregate (abs. values)	0.16 ± 0.17	3.5 ± 3.0	0.50 ± 0.09	0.48 ± 0.27	0.43 ± 0.18	6.0 ± 7.0	0.51 ± 0.15	0.49 ± 0.31

*Indicates metrics that had a statistically significant change over the rehabilitation program (1 for the PPIT, 4 for the VPIT). Aggregate indicates the median ± inter-quartile range of the absolute (abs.) value across metrics

For concurrent validity in pwMS (Table 2), no significant differences between VPIT and PPIT were found for both BBT and NHPT.

### Usability

The median of the SUS for able-bodied participants was 90 ± 15 for the PPIT, 85 ± 14.4 for the VPIT, and 70 ± 15 for the VPIT-2H (Fig. 3E). A Friedman omnibus test revealed a significant difference between the three conditions (p < 0.001). Post-hoc tests revealed no statistical difference between the SUS of PPIT and VPIT but showed significant lower scores for VPIT-2H than PPIT (p < 0.001) and VPIT (p < 0.001).

The median of the SUS for pwMS was 90 ± 12.5 for the PPIT and 82.5 ± 22.5 for the VPIT, which was a statistically significant difference (p < 0.01, Fig. 3E).

## Discussion

Technology-based upper limb assessments can provide digital health metrics that are expected to expand on the limitations of conventional clinical scales and serve as novel, sensitive, and objective endpoints for clinical trials evaluating pharmacological or rehabilitation interventions in neurological disorders [3, 5–7]. Such assessments often rely on 2D VR environments and tasks with different levels of complexity, two factors that were shown to have influence on movement kinematics [21, 22]. Herein, we aimed to additionally describe which influence these factors have on the clinimetric properties of digital health metrics in terms of test–retest reliability, measurement error, responsiveness, and concurrent validity. As a secondary aim, we strived to evaluate the influence of virtual environment and task complexity on the values of the metrics, the intra-participant variability, and the usability of the assessment platform. For this purpose, we compared a previously validated technology-based assessment, the VPIT, with two newly designed tasks based on the VPIT concept, the VPIT-2H (VR environment with reduced task complexity) and the PPIT (physical task with reduced task complexity).

### Aspects of movement kinematics and kinetics differ between assessment setups and tasks

In line with previous research, our results show a considerable influence of the different assessment setups and tasks. Specifically, goal-directed movements were smoother and faster in the PPIT than the VPIT and VPIT-2H. This likely results from the end-effector and task being co-located in the PPIT, whereas depth perception and a visuomotor transformation from the end-effector space to the VR space is required for the VR-based tasks [40]. Additionally, this might be influenced by faster movements being shorter and having less data samples, which in turn may affect the calculation of smoothness metrics. Goal-directed movements were more efficient in the VPIT than the VPIT-2H and PPIT, which might be an artifact of the wall in the center of the pegboard that was introduced in the latter two conditions. This wall is supposed to hinder dragging the peg across the pegboard (i.e., performing movements only in the horizontal plane), which is indeed more efficient than performing three-dimensional movements but was not desired when designing the assessment task initially [24, 41]. Even though grip force control was smoother in able-bodied participants for the PPIT, these results were not confirmed in pwMS.

Overall, these findings support previous research showing reaching movements in VR are typically less smooth and slower compared to physical environments, and that increasing task complexity decreases movement smoothness and speed [18, 20–22, 42, 43].

### Physical assessment task with low task complexity has lowest intra-participant variability

The main contribution of our work is to evaluate whether clinimetric properties of metrics describing movement kinematics and kinetics are improved by an instrumented physical assessment task, compared to a haptic VR-based task, and by tasks with different complexity levels. Given that many clinimetric properties are complex statistical constructs that are influenced by multiple measurands, we performed an intermediate analysis step describing the metrics’ intra-participant variability. This is an easily quantifiable construct, based on the coefficient of variation, and is expected to have a strong influence on the clinimetric properties [37, 38].

Indeed, we observed that a physical instrumented task with the expected lowest complexity consistently had the lowest intra-participant variability for almost all digital health metrics when compared to a similar haptic VR-based task with higher complexity (VPIT), in both able-bodied participants and pwMS. Additionally, the physical instrumented task with low complexity showed lower intra-participant variability for movement speed and aspects of movement smoothness as compared to the haptic VR-based task with low complexity in able-bodied participants. This suggests that the biggest contribution to increased intra-participant variability stems from the VR environment, including the required visuomotor transformation to map between end effector and display as well as the requirements for depth perception on a 2D computer screen. This is in line with previous work showing that the required visuomotor transformation has a strong influence on task performance in exoskeleton-based reaching, especially when learning the task initially [40]. Further, the increased intra-participant variability for the haptic VR-based task with higher complexity likely stems from the larger possible choice in strategy (e.g., order of pegs) and difference in movement trajectories across different peg-hole combinations and task repetitions [22]. Additionally, tasks with increasing complexity are known to have additional cognitive demand [44], which can also alter repeated task performance.

### Clinimetric properties are not significantly different between assessment setups and tasks

These strong changes in the metrics’ intra-participants variability across task conditions did not lead to systematic changes in most of the metrics’ clinimetric properties. While the PPIT metrics indeed had the smallest measurement error compared to the VPIT and VPIT-2H metrics, these differences were not statistically significant and test–retest reliability of the metrics was mostly similar across task conditions. The main difference between the conditions was in the responsiveness in pwMS, where the VPIT metrics actually achieved significantly better performance than the PPIT.

These findings are surprising, given the strong effects on the intra-participant variability level, and that intra-participant variability is factored into the calculation of test–retest reliability and measurement error. Most likely, this is because the inter-participant variability also increases in tasks with higher complexity and a VR environment. This allows to compensate for the increased intra-participant variability in the calculation of the intra-class correlation coefficient, thus allowing to maintain high levels of test–retest reliability as metrics are still able to accurately discriminate between participants [38]. Indeed, inter-participant variability was consistently increased for the VPIT compared to the PPIT (e.g., Figure SM3 and SM4), thereby supporting this argumentation. The reason why this increased level of intra- and inter-participant variability in the VPIT metrics led to higher responsiveness compared to the PPIT metrics might be that increased inter-participant variability indicates that the metric is more responsive to behavioral changes, not only between participants but also because of an intervention.

While the absolute levels of test–retest reliability and measurement error for the VPIT metrics were not excellent, this was expected given that they were estimated in able-bodied participants that are known to have less inter-participant variability, which influences the calculation of the intra-class correlation coefficient. This is supported by VPIT-based test–retest studies in persons with neurological disorders that achieved considerably better reliability [9, 25].

The absolute level of responsiveness of the digital health metrics in pwMS is challenging to interpret given the lack of comparable literature and the need for a dedicated analysis in a larger sample. A careful initial evaluation based on commonly used cut-offs to judge effect sizes would suggest that the responsiveness of the VPIT metrics was superior to the PPIT, NHPT, and BBT (details in SM and Table SM2 and SM3). Specifically, the VPIT showed small to moderate effects that were significant for four metrics and, for example, the VPIT SPARC RT metric that indicated 10 pwMS improved movement smoothness beyond measurement error. In comparison, the NHPT showed only non-significant small effects and only three individuals improved beyond measurement error in NHPT. For the BBT, moderate effect sizes were observed and three pwMS improved beyond measurement error, albeit missing data challenges the comparability. Also, all pwMS that had improvements above the SRD in the BBT or NHPT also improved above the SRD in at least one metric in the VPIT and, except for one pwMS, also in the PPIT. Hence, in addition to larger effect sizes, the kinematic and kinetic metrics identified pwMS that improved in movement quality or grip force control but did not improve according to the clinical scales. However, these findings require further investigation in carefully designed, larger interventional studies that can highlight the value of digital health metrics in the absence of a true clinical ground truth.

### Concurrent validity in pwMS is similar across tasks

Interestingly, the changes in intra- and inter-participant variability across task conditions did not significantly influence concurrent validity in pwMS. This is surprising, given that movements were most natural in the physical instrumented task, according to the metrics describing smoothness and speed, and should therefore more closely resemble the conventional assessments. This suggests that sensorimotor impairments can be captured by digital health metrics from behavioral tasks that elicit different levels of movement smoothness and speed.

### Instrumented assessment with a haptic VR or physical task are both suitable for clinical studies

These findings highlight that both haptic-based assessment with 2D VR environments and physical task-based instrumented assessments can provide metrics with adequate clinimetric properties for potentially providing novel endpoints for clinical studies. However, the haptic VR-based assessment with high task complexity had higher responsiveness in pwMS, thus being most promising for longitudinally assessing pwMS, even though these preliminary results need further confirmation. This supports the usage of haptic VR-based assessments with different levels of complexity that are already widely present in the research community [11–15]. It is also important to highlight that the physical instrumented task with low task complexity was superior in certain aspects to the VR-based tasks. Specifically, in the PPIT, movements were most intuitive (i.e., had highest speed and smoothness), intra-participant variability was lowest and usability ratings in pwMS were highest. This highlights that a physical instrument task with low task complexity might also be beneficial in certain clinical use-cases.

### Assessment design should consider usability, minimize intra- and maximize inter-participant variability

Furthermore, these results highlight that minimizing intra-participant variability should not be the main criteria when designing an assessment with optimized clinimetric properties. Instead, the relationship of intra-participant to inter-participant variability needs to be considered and to obtain ideal clinimetric properties, intra-participant variability should be minimized whereas inter-participant variability should be maximized. While this is already obvious from the basic definition of reliability, previous research suggested indeed that the main focus should be on minimizing intra-participant variability to optimize the responsiveness of an assessment [37]. While minimizing intra-participant variability can be achieved by reducing task complexity and switching from a haptic VR to a physical task, it is an open question how assessment tasks should be designed to additionally maximize inter-participant variability. Potentially, this could be achieved when participants have disability-related differences in their behavior when performing the assessment, but consistently stick to that behavior throughout repetitions. While this can potentially be implemented by designing complex assessment tasks, it is most important that the task complexity is also catered to the disability level of the target population. Specifically, persons with severe motor or cognitive disabilities would not be able to perform complex tasks and instead need simpler alternatives. Also, it needs to be considered that increasing task complexity challenges the dissociation of different mechanism underlying abnormal task performance. For example, an added visuomotor transformation in a sensorimotor task could make it more difficult to distinguish the effect of sensorimotor and visuomotor impairments on task performance. This highlights that task complexity is closely linked to the interpretability of digital health metrics.

As not only clinimetric properties of metrics but also the usability of the assessment platform needs to be considered when attempting to establish a technology-based assessment, we asked participants to rate the platforms usability based on the SUS. While the usability of all platforms was rated as acceptable, the PPIT had the highest usability for able-bodied participants and pwMS. This, together with the objective data showing smoother and faster movements in physical environment than VR, confirms our initial hypothesis that movements in the physical environment are perceived as more natural than in VR. This indicates that using a physical instrumented task instead of a haptic VR-based task might help to further increase the usability of a technology-aided assessment platform.

Next to the assessment design, the definition and validation of suitable digital health metrics is of crucial importance to enable clinical integration. Our work considered a large set of 10 metrics, which were identified from an initial set of 77 candidate metrics through a systematic selection procedure and deemed as most reliable and relevant for the VPIT [9, 10]. Hence, our analysis provides an understanding of the effect of task complexity and VR on a representative set of kinematic and kinetic metrics describing behavior during goal-directed tasks. This serves as a foundation to further select and validate a single metric or a composite score that would be most meaningful to address specific clinical research questions.

### Limitations

Even though the different assessment tasks are based on the same device and have similar task dimensions and number of movements, there are still slight differences, for example, in terms of visual feedback related to the level of grasping force, the presence/absence of the wall requiring increased lifting during movements, and the difference between the haptic and actual physical feedback. These changes might have had an undesired influence on the assessment outcomes. Clearly, the presented results are specific to the common use case of VR-environments presented on a 2D computer screen and might not generalize to other setups using collocated VR or head mounted displays [7, 20, 45]. Also, our definition of task complexity was derived from the concept of component complexity and focused especially on the number of available objects and the absence/presence of a predefined order in that the tasks needs to be completed, thereby capturing the level of motor and cognitive processing involved in a task [44]. It remains to be explored whether similar results would be obtained when manipulating other aspects of task complexity, for example in terms of the number of joints that are involved in a goal-directed movement. Additionally, it would have been interesting to explore a potential relationship between cognitive abilities and differences in performance between task conditions, but such data was not available in the context of this study. Lastly, the SRD values needed for the responsiveness analysis were based on a young able-bodied population that was not age-matched to the population of pwMS. While this does not affect the comparison of responsiveness across conditions, one should treat the magnitude of the number of pwMS exhibiting changes larger than the SRD with appropriate caution.

## Conclusion

Our work provides evidence that both a technology-based assessment in a physical environment with low task complexity and a haptic VR-based assessment with low or high task complexity provide digital health metrics with adequate clinimetric properties. However, the haptic VR-based assessment had superior responsiveness, thus being preferable when longitudinally assessing pwMS. In contrast, the physical instrumented task had higher usability in pwMS, thus being potentially more suitable for clinical use. These findings emphasize that different clinical application might benefit from different technology-based assessments.

Also, our work highlights the importance of taking both intra-participant and inter-participant variability into account when designing technology-based assessments with optimal clinimetric properties, which should be considered jointly with the usability of an assessment platform. Overall, this work provides novel insights that can inform the design and choice of technology-based assessments with optimal clinimetric properties and usability. This is urgently needed to ensure digital health metrics fulfill their expectations in clinical research and practice.

### Supplementary Information

Supplementary Material 1.

## Data Availability

The data from this study are available upon reasonable request and under consideration of the data sharing agreement of the study.
