# Supplementary material for: Influence of virtual reality and task complexity on digital health metrics assessing upper limb function
Source: J Neuroeng Rehabil. 2024 Jul 27;21:125. doi: 10.1186/s12984-024-01413-x (PMC11282591; doi:10.1186/s12984-024-01413-x)
Supplement: Supplementary file 1 — Supplementary Material 1. [file 12984_2024_1413_MOESM1_ESM.docx]

**SUPPLEMENTARY MATERIALS**

**Title: Influence of virtual reality and task complexity on digital health metrics describing upper limb function**

**Authors:**

Christoph M. Kanzler^1,2^, Tom Armand^1^, Leonardo Simovic^1^, Ramona Sylvester^3^, Nadine Domnik^1^, Antonia M. Eilfort^1^, Carola Rohner^1^, Roger Gassert^1,2^, Roman Gonzenbach^3^*, Olivier Lambercy^1,2^*

*authors contributed equally.

1 Rehabilitation Engineering Laboratory, Institute of Robotics and Intelligent Systems, Department of Health Sciences and Technology, ETH Zurich, Zurich, Switzerland.

2 Future Health Technologies, Singapore-ETH Centre, Campus for Research Excellence And Technological Enterprise (CREATE), Singapore.

3 Rehabilitation Center Valens, Valens, Switzerland.

**Corresponding author:** Christoph M. Kanzler, [relab.publications@hest.ethz.ch](mailto:relab.publications@hest.ethz.ch)

**Results**

For the NHPT, the SRM was 0.18, the change across the rehabilitation program was not statistically significant on a group level (p>0.05), and three individuals (i.e., 14.3% of analyzed sample) improved in the NHPT above the measurement error (SRD_NHPT_=5.32s, Lamers et al. 2014; individual improvements 75.9s, 33.1s, and 13.3s). For the BBT (13 analyzable patients), the standardized response mean was 0.62 and three individuals (i.e., 23.1% of analyzed sample) improved in the BBT above the measurement error (SRD_BBT_=8.11 blocks/min, Lamers et al 2014; individual improvements 10, 9, and 11 blocks/min). Statistical testing of the change in BBT was avoided due to missing data. All pwMS that had clinical improvements above the SRD also improved in at least one VPIT metric. For the PPIT, all pwMS that had clinical improvements above the SRD, except pwMS with ID 20, also improved in at least one metric. As expected, 10 pwMS (PPIT) and 12 pwMS (VPIT) exhibited improvements at least one metric, even though the pwMS did not show clinical improvement above the SRD.

Figure SM1 Visualization of digital health metrics for able-bodied participants and the three different task conditions. TP: Transport. RT: Return.

Figure SM2 Visualization of digital health metrics for able-bodied participants and the three different task conditions (continuation of Figure SM1).

Figure SM3 Visualization of digital health metrics for persons with MS and the two different task conditions.

Figure SM4 Visualization of digital health metrics for persons with MS and the two different task conditions (continuation of Figure SM3).

Figure SM5 Test-retest reliability in terms of intra-class correlation coefficients (ICC) for the able-bodied participants.

Table SM1 Smallest real difference (SRD) values for all metrics extracted from the PPIT, VPIT, and VPIT-2H. SRD% denotes the SRD normalized with respect to the range of a metric. TP: transport. RT: return. SPARC: spectral arc length.


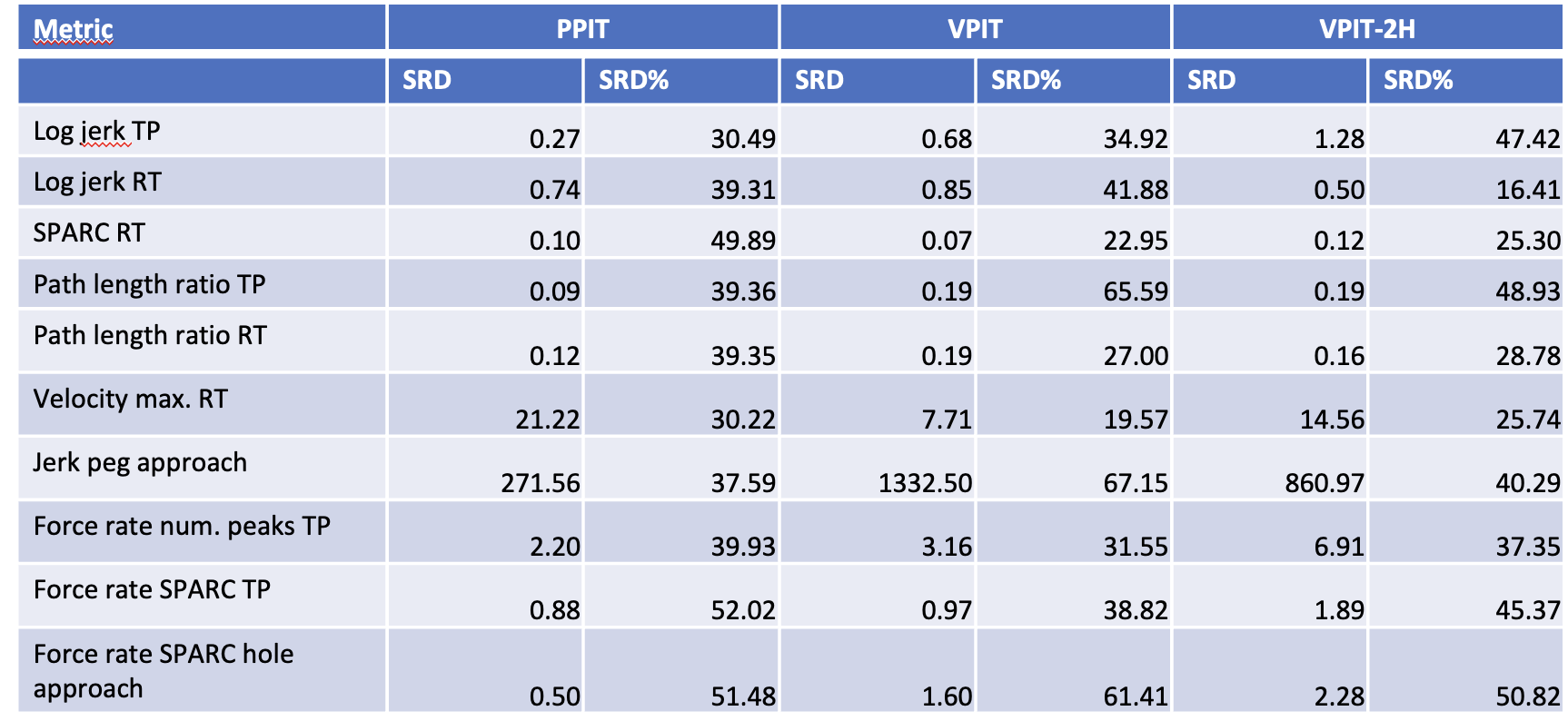


Table SM2 PwMS that exhibited improvements in NHPT or BBT above the measurement error. The instrument that improved above measurement error is highlighted in bold font. IDs of pwMS were arbitrarily defined.

| PwMS ID | NHPT (pre; post) in s | BBT (pre; post) in blocks/min |
| --- | --- | --- |
| 5 | **160.0; 84.1** | 28; 31 |
| 8 | **88.6; 55.4** | 39; n.a. |
| 16 | 23.1; 24.1 | **55; 65** |
| 18 | 31.6; 45.5 | **46; 55** |
| 19 | **107.6; 94.3** | 18; 22 |
| 20 | 23.0; 22.0 | **59; 70** |

Table SM3: Number of PPIT and VPIT metrics that improved above the SRD for each PwMS. Bold font indicates the pwMS that also increased in BBT or NHPT above the SRD. Only pwMS with completely available pre- and post data were considered.

| PwMS ID | #PPIT metrics improved above SRD | #VPIT metrics improved above SRD |
| --- | --- | --- |
| 1 | 1 | 1 |
| 4 | 2 | 2 |
| **5** | 3 | 2 |
| 6 | 4 | 1 |
| 7 | - | - |
| **8** | 4 | 1 |
| 9 | 1 | 6 |
| 10 | - | 5 |
| 11 | 3 | 5 |
| 12 | 1 | - |
| 13 | - | 4 |
| 14 | - | 2 |
| 15 | 1 | 3 |
| **16** | 2 | 3 |
| 17 | 5 | 3 |
| **18** | 3 | 3 |
| **19** | 4 | 4 |
| **20** | - | 3 |
| 21 | 1 | 1 |
| 22 | - | - |
| 23 | 2 | 4 |
